# Using Online 24-h Dietary Methodology to Validate the Psychometric Properties of a Dietary Scoring Tool with an International Sample of Adults Living with Multiple Sclerosis

**DOI:** 10.3390/nu14214568

**Published:** 2022-10-31

**Authors:** Vivienne Guan, Steve Simpson-Yap, Nupur Nag, George Jelinek, Sandra Neate, Yasmine Probst

**Affiliations:** 1 School of Medical, Indigenous and Health Sciences, University of Wollongong, Northfields Avenue, Wollongong 2522, Australia; 2 Illawarra Health and Medical Research Institute, Northfields Avenue, Wollongong 2522, Australia; 3 Neuroepidemiology Unit, The University of Melbourne, Grattan Street, Parkville 3010, Australia; 4 Menzies Institute for Medical Research, University of Tasmania, 17 Liverpool St., Hobart 7005, Australia

**Keywords:** multiple sclerosis, dietary intake, diet score, dietary pattern, dietary assessment

## Abstract

Understanding the dietary characteristics of people living with multiple sclerosis (plwMS) may assist in the planning of nutrition interventions for multiple sclerosis; yet dietary assessment methods in existing studies are not well established. The aim of this study was to validate the psychometric properties (construct validity and internal consistency) of the Dietary Habits Questionnaire (DHQ) against repeated online 24-h recall dietary assessments. The DHQ is a 24-item tool that is scored using ten dietary sub-scores. Total DHQ scores can range from 20–100 and are considered indicative of the quality of dietary intake with higher scores reflecting increased quality. People living with a relapsing-remitting MS phenotype who had completed a modified DHQ were recruited from the international Health Outcomes and Lifestyle In a Sample of people with Multiple sclerosis (HOLISM) cohort. Repeated 24-h recall via the online Automated Self-administered Assessment-24 (ASA-24) tool were modelled to reflect usual dietary intakes using the Multiple Source Method. DHQ scores of eight sub-scores: three key nutrients, three food groups and two food preparation practices, were calculated and statistically compared with ASA-24 usual intake data. Principal component analysis of the ASA-24 data was undertaken to understand dietary patterns of the sample. Of the 105 participants, valid 24-h recall data were available for 96 plwMS (n = 66 1 day, n = 30 ≥ 2 day). The median total DHQ score was 84.50 (IQR: 77.04, 91.83) points. The highest absolute correlations were between the DHQ scores and ASA-24 data for cereal (r = 0.395, *p* < 0.001), fruit and vegetables (r = 0.436, *p* < 0.001), and total dietary fiber (r = 0.482, *p* < 0.001). Five dietary patterns emerged from the data explaining 42.12% variance and reflecting exposure of plwMS to the influence of ‘MS diets’. The DHQ appears to be appropriate for screening participants with relapsing-remitting MS. Evidence-based dietary models focusing on food are required to monitor the quality of an overall dietary pattern and set priorities for the planning nutrition interventions for plwMS.

## 1. Introduction

Multiple sclerosis (MS) affected 2.8 million people globally in 2020, which is 30% higher than in 2013 [1]. MS is a chronic inflammatory neurodegenerative autoimmune disease affecting the central nervous system, that can adversely affect bodily functions leading to significant impairment, health burden and disability [2]. Depending on the acute demyelinating lesion location and the extent of the inflammatory process, people living with MS (plwMS) may experience fully or partially reversible episodes of neurological deficit, usually lasting days or weeks, characterized by a spectrum of symptoms [2].

Disease-modifying treatments can stabilize disease progression in the initial relapse-remitting phase of MS, and potentially delay transition to a secondary progressive phase of the disease [2]. However, treatments often have many side effects [2]. Thus, plwMS look for alternatives to disease management, such as dietary interventions. PlwMS have reported perceiving nutrition and many specific dietary components, such as meat, fat and sugar to have an impact on their MS disease activities [3], though the evidence is inconclusive. Mechanistic studies for MS in vitro and in vivo suggested that some nutrients, such as saturated fats, polyunsaturated fatty acids, added sugars, and dietary fibers, may contribute to the process of inflammation [2,4]. However, an umbrella review found that the single-nutrient approach applied in randomized controlled trial studies for MS, had limited effects on MS progression including rate of relapse, disability, occurrence of new brain or spinal lesions, and associated symptoms such as fatigue [5]. This suggests that studies focused on dietary patterns are needed to grow the evidence for diet-disease relationships to support MS management.

Dietary patterns that are high in whole foods including fruits, vegetables, legumes, and whole grains, and low in added sugars, have been associated with lower levels of disability and less severe rates of depression as a symptom of MS [6,7]. While these results are suggestive, it is increasingly the consensus in dietary research, including in MS, that broad dietary patterns or the quality of a diet are more appropriate approaches than individual foods or nutrients, as dietary intake acts as a combined construct with holistic effects. 

PlwMS are exposed to a range of ‘MS diets’ that are suggested for disease management, often supported by limited scientific evidence. Common dietary approaches among plwMS include the Swank diet, a low saturated fat, high fiber pattern that recommends a high vegetable and low/no meat approach, the Paleolithic (paleo) or modified paleo Wahl’s diet, which recommends limited grains, high intake of vegetable of particular forms, and high grass-fed meat and wild fish and the Overcoming MS (OMS) approach, which suggests limited meat and dairy, with intakes of grains, vegetables, fruit, dairy alternatives, fish and seafood, and the Mediterranean diet which emphasizes fruits, vegetables, eggs, whole grains, fish and seafood, legumes, nuts, olive oil and lean red meat [8,9]. Currently, there are no dietary guidelines for people with MS; and therefore, a balanced diet that is applicable to the general population is recommended in the absence of conclusive evidence [4,10]. Thus, clinical studies focused on the whole of diet appraoch are required to complement the evidence for the diet-disease relationships.

Diet quality scores, or indices, serve as useful tools to evaluate the healthiness of a diet and are used to monitor quality or to evaluate the effects of dietary interventions [11]. These tools are usually developed from healthy dietary patterns derived from evidence-based dietary guidelines for the general population, or for the prevention of a specific disease often through *a priori* methods [12]. Alternatively, data-driven or *a posteriori* methods can be used to identify dietary patterns from intake data [11]. For example, principal component analysis derives dietary patterns based on the interrelationships between dietary variables (e.g., food groups) [11]. The derived dietary patterns can then be converted to a diet quality score, which can be used to explain variation in the dietary intakes of a population. Data-driven approaches are more likely to be applied for nutrition education and the planning of nutrition interventions to slow disease progression. 

Studies capturing the health and outcomes of plwMS at times include measures of food intake amidst other lifestyle factors. An example of this, is the international Health Outcomes and Lifestyle In a Sample of people with Multiple sclerosis (HOLISM) longitudinal study [13]. The HOLISM study provides data for participants in different countries using consistent approaches to data collection. The HOLISM study uses participant responses to the Dietary Habits Questionnaire (DHQ) [14] to derive a score, finding associations between high scores and positive health outcomes [15,16,17,18]. The DHQ was developed based on the Dietary Guidelines for Australian adults and the nutrition recommendations of the National Heart Foundation of Australia [19] but has not been validated for plwMS. Evaluating diet scores such as the DHQ provides evidence on whether a score can adequately describe the dietary pattern that they are designed to measure [12]. Therefore, the aim of this study was to validate the psychometric properties (construct validity and internal consistency) of the DHQ against repeated online 24-h recall dietary assessments. Given the lack of evidence-based dietary guidance for people with MS, data-driven dietary patterns were also derived to determine the variability of dietary intakes within the population.

## 2. Materials and Methods

### 2.1. Study Population

Participants of this study were recruited from the HOLISM cohort, which includes self-complete online questionnaires every 2.5 years related to sociodemographic, clinical, and lifestyle characteristics. Between May and August, 2012, the HOLISM study recruited participants using online platforms for plwMS. A sample of 2239 responses were established at baseline [18]. All HOLISM study participants were 18 years old or above and had a self-reported a physician diagnosis of MS. 

For this sub-study, participants living with relapsing-remitting MS (RRMS) upon entry to the HOLISM study, who had responded to questionnaires at the 5-year review (most recent data collection at the time of the sub-study), and were residing in Australia, New Zealand, the United States of America (US), Canada, the United Kingdom (UK) and Ireland were included. No formal sample size was calculated. For dietary validation studies, without a biomarker reference, a sample of between 100–200 participants is recommended [20]. For Australia (n = 232), the UK (n = 89), and US (n = 154), a random selection of cases using random sequence generation by a researcher independent of the analyses were invited to participate; all participants for New Zealand (n = 70), Canada (n = 22) and Ireland (n = 16) were included due to the small samples. The participants were also limited to those with a Patient Determined Disease Steps (PDDS) score of ≤6 (normal to bilateral support). PDDS is a self-reported tool of perceived disability that is commonly used with plwMS [21]. Participant data about sociodemographic (age, sex, location, height, weight), lifestyle (dietary habits, smoking and alcohol use, sunlight and vitamin D, exercise, meditation), and clinical characteristics (disease activity, fatigue, depression, medication use) were extracted from the HOLISM data, as described previously [13,22]. 

### 2.2. Measurements

#### 2.2.1. Dietary Intake Assessment and Dietary Intake Data

The 24-h recall dietary assessment method was selected as the reference method for the validation undertaken in this study, due to its reduced cognitive burden as it only requires recall of the previous day of intake guided by individualized prompts. Participants were asked to complete two online 24-h recalls capturing a week and weekend day of intake, where possible. The Australian version of the Automated Self-administered Assessment (ASA)-24-AU tool [23] was used to obtain dietary intake data from the participants residing in Australia, New Zealand and the UK. Data analysis of the ASA-24-AU is automated using the Australian Food, Supplement, and Nutrient Database for Estimation of Population Nutrient Intakes 2011–2013 (AUSNUT 2011-13) [24]. 

The equivalent ASA-2018 tool was used to collect dietary intake data of the US and Canadian participants. The ASA-24-AU and ASA-2018 tools are both based on the validated US Department of Agriculture (USDA) automated multiple-pass methodology [25]. To standardize analyses, the food items derived from the ASA-2018 were aligned to the food groups of the AUSNUT2011-2013, following a published protocol [26]. In brief, the food items from the USDA food composition database, which underpins ASA-2018, were matched to the AUSNUT 2011–2013 database, which underpins ASA-24-AU, using food matching guidelines produced by INFOODS Food and Agriculture Organization [27]. Food matches were created based on conceptual and nutrient similarities, where possible. 

Intakes of the nested hierarchical food groups of the AUSNUT 2011–2013 food classification system were then calculated for each participant for comparability with the DHQ sub-scores. 

Intakes of food groups from the Australian Dietary Guidelines were assessed. The Australian Health Survey—Australian Dietary Guidelines (ADG) food composition database [28] was used to calculate intake (serves per day) of the five food groups (vegetables, fruits, grains, milk and milk alternatives, and lean meat and meat alternatives) and intakes of discretionary foods (high in saturated fat, added sugars, or both) [19] based on a published method [29]. For this process, the ADG database was applied to the food items reported using the AUSNUT 2011–2013 food ID codes. This allowed for food items that are a composite of a number of foods (ingredients) to be deconstructed into their component items. Food groups and their examples are presented in Supplementary Table S1. 

Discretionary food items were identified using the AUSNUT 2011–2013 Discretionary Food List [30]. One serve of a discretionary food or alcoholic beverage was defined as containing 600 kJ or 10 g alcohol, respectively [19]. The multiple source method (MSM) regression model [31] was used to convert the reported intakes to usual intake data, for total energy (kJ), nutrients and food groups. The MSM applies a two-part data shrinkage approach to dietary intake data to adjust the 24-h recall intake data to habitual intake data. For this to occur, a portion of the intake data in the model needs to be repeated, i.e., repeated 24-h recalls. 

#### 2.2.2. Diet Habits Questionnaire

The DHQ is the primary tool used to capture dietary data for two large national MS cohorts [7,32]. Diet was queried using a modified version of the DHQ dietary screening tool, omitting questions regarding sodium intake and alcohol, as described previously [13]. Sodium and alcohol data was not collected in the HOLISM study due to issues with the specificity of the outcomes; the data was, therefore, not available for this sub-study. In brief, the DHQ is a 24-item tool that is scored based on categorical responses, in this study, using eight dietary sub-scores (the unmodified tool includes ten sub-scores). Total DHQ scores range from 20–100, calculated using a scoring proforma. Scores are considered indicative of the quality of intake with higher scores reflecting increased quality [14]. High fiber breakfast cereal, wholemeal or whole grain bread, other cereals, legumes, raw nuts and seeds, reduced-fat or low-fat milk/yoghurt/cheese, fish, processed meat, pastries/cake/sweet biscuits/croissants, and take-away foods were reported as frequency options ranging from less than once per week or never to six or more times per week or always. Categories for less frequent consumption receive a lower score and more frequent consumption a higher score (1 to 5 points), with scores reversed for discretionary items. Fruit and vegetables were reported as servings per day (<1 piece for 1 point, two or more pieces for 5 points). Types of vegetables per day were queried with five options ranging from none up to five or more types (1 point each). Types of spreads on breads or cracker biscuits, salad dressings, cooking sauces, and cooking fats were also queried (solid fats for 1 point to unsaturated fats for 5 points). Food preparation methods were queried including steaming, poaching, microwaving, casseroles, grilling, stir fries, dry roasting (5 points), roasting in fat, shallow frying and deep frying (1 point). 

#### 2.2.3. Other Measures

Sex was queried as male/female/other. Age was calculated from self-reported birth date. MS phenotype was queried as benign/RRMS, secondary progressive MS, primary progressive MS, and other/not sure. Participants reported the date of MS onset and MS diagnosis, from which durations from MS onset and diagnosis were calculated. Body Mass Index (BMI) was calculated from self-reported height (meters) and weight (kilograms) and classified as per the World Health Organization categories of underweight (<18.5 kg/m^2^), normal weight (18.5–24.9 kg/m^2^), overweight (25.0–29.9 kg/m^2^), and obese (≥30.0 kg/m^2^) [12].

### 2.3. Statistical and Data Analysis

Due to the small number of participants per region, this study reports the combined data for the validation and dietary pattern analyses. Demographic and general intakes have been reported by country due to the use of different dietary assessment tools. Statistical analyses were conducted using IBM SPSS Statistics (version 25.0, IBM Corp, Chicago IL, 2017). A two-sided *p* < 0.05 was considered statistically significant. Normality of all data were assessed using the Shapiro–Wilks test. Continuous data are presented as mean (standard deviation) and median (interquartile range) for parametric and non-parametric data, respectively.

To evaluate the DHQ construct validity, DHQ sub-scores were examined to determine the relationships with corresponding nutrient/food group data obtained from the MSM output for the ASA-24 data using Spearman’s rank-order correlations. Independent samples *t*-tests were conducted to determine if there were differences for those who reported 1 recall vs. 2+ recalls. For the nutrient data of the DHQ, Bland–Altman plots were unable to be constructed for the DHQ sub-score and the ASA-24 usual intake data due to differences in the units of measurement (score vs. grams per day). 

The distributions of dietary intake data were also examined for the variation among participants. As energy intake is not evaluated in the DHQ, no energy adjustments could be made. Quartiles of DHQ total scores were calculated, where Q1 indicated a diet least consistent with national recommendations and Q4 the most consistent with national recommendations. Trends across the DHQ total score quartiles (dependent variable) with each DHQ questionnaire scores (independent variable), intake of food groups (independent variable), energy (independent variable) and nutrients (independent variable) were assessed using linear regression [33]. 

To compare DHQ total scores by sex, an independent *t*-test for parametric data, and the Wilcoxon-Mann–Whitney test for non-parametric data were used. For age (middle adulthood 25–44 years, older adulthood 45–64 years and average retirement >65 years) group data, one-way analysis of variance with Bonferroni correction was used [34]. 

To evaluate internal consistency, DHQ total scores were examined to determine the relationships with sub-score categories using Spearman’s rank-order correlations. Correlation coefficients in the range of 0.4–0.6 suggest a that a dietary assessment instrument is reliable [35]. The Cronbach’s coefficient alpha of DHQ total score and the sub-scores were used to evaluate the degree to which each component influenced the total score [33]; >0.7 suggested adequate internal consistency [36]. The standardized Cronbach’s alpha for the HOLISM sub-sample in this study was also assessed to capture the systematic variation underlying the DHQ components. 

To understand the dietary intakes of the plwMS in the sample, a data-driven method, principal component analysis (PCA), was performed. As not all participants provided two days of recall, the PCA was conducted based on one single day consumption data of the AUSNUT 2011–13 major food groups (grams per day, Supplementary Table S1) [24]. Our primary interest was in dietary patterns based on the relative composition of the diet, and the total amount consumed. No energy adjustments were made for the intake data. The suitability of data for PCA was assessed prior to analysis. Sampling adequacy was supported by a Kaiser-Meyer-Olkin measure of 0.5 and Bartlett’s test of sphericity 0.004, indicating appropriate data. Inspection of the anti-image matrices was performed to determine the food groups to retain for PCA. For this, eigenvalues >1.0 were considered along with examination of the scree plot. To simplify the structure and improve the interpretability, an orthogonal (varimax) rotation was applied. Calculation of factor scores for each component was performed using the regression method. Food groups with absolute factor loadings >0.2 were considered significant contributors to the dietary patterns with each food group only considered once for each pattern based on highest, lowest and meaningful attributions. 

## 3. Results

### 3.1. Characteristics of the Study Participants

Across the six countries of the HOLISM study, included here, 238 participants were invited to join this study (Supplementary Figure S2). Of these, 105 participants attempted to complete an ASA-24 recall, with nine participants not completing a valid recall of a 24-h period of meals and, thus, were excluded from the analysis. Therefore, a total of 96 participants (17 male, 79 female) were included in the analysis (Table 1). The median time since MS onset was 8.5 years and time since diagnosis, 3.9 years. In total, 69% of participants (n = 66) were of a normal weight and 10% an obese weight BMI category (n = 10). 

### 3.2. Intake of Nutrients and Food Groups 

Sixty-six participants completed one 24-h recall and 30 participants completed two or more 24-h recalls (Table 2). Both participants who completed 3 recalls had a first recall incomplete followed by two valid recalls. Of those who completed two recalls with one invalid day, their data was considered as one recall. Mean total daily energy intake was 8.1 ± 2.1 MJ. There was no significant difference in reported energy intake between those who completed one versus two recalls (*p* = 0.965). Reported intakes of food groups were aligned with the recommendations of the Australian Dietary Guidelines and fiber intake exceeded the Adequate intake nutrient reference value recommendation (34.1 g) when considering ASA-24 data. 

### 3.3. Evaluation of the DHQ

#### 3.3.1. Construct Validity

Relative validity outcomes for the three nutrient components of the DHQ with the ASA-24 data provided weak associations for total fat (r = −0.086, *p* = 0.404) and moderate associations for dietary fiber (r = 0.505, *p* < 0.001) and omega-3 fats (r = 0.512, *p* = <0.001).

The median total DHQ score was 84.5 (IQR: 77.0, 91.8). (Table 2). Higher DHQ score quartiles were associated with higher intake of vegetables and fruits (*p* = 0.005), protein (*p* = 0.397), total polyunsaturated fatty acids (*p* = 0.006), potassium (*p* < 0.001), magnesium (*p* < 0.001), vitamin C (*p* < 0.001) and total folate (*p* = 0.001). 

However, when food group data from the ASA-24 were compared with DHQ sub-score quartiles, variability was seen for vegetables, cereals and discretionary items. It was expected that a linear increase would be identified whereby higher total DHQ scores (higher quartiles) would reflect higher amounts of intake as was seen for the fruit data (Table 3).

Female (median total DHQ scores 85.5, IQR: 76.0–91.8) and male (median total DHQ score 85.5, IQR: 79.6–90.5) participants had similar DHQ total scores, though females had significantly higher sub-scores for omega-3, food choices, and food preparation than males (Table 4). The intakes of total daily energy, cereal, meat and alternatives, protein, carbohydrate, total fatty acids, total monounsaturated fatty acids, total dietary fiber, magnesium, potassium and total folate were significantly higher for male than female participants (*p* < 0.05). 

Intakes across the age groups were significantly different, with middle adulthood participants having a mean score of 86.8 (IQR 77.0, 92.1) points and young adults a median score of 79.6 (IQR 74.0, 85.8) points, driven by higher scores for cereal, fruit and vegetables, lower scores for takeaway foods, fiber, fats, omega-3, food choices, and food preparation techniques (Table 4). The older age group had higher intake of vegetables than the younger age group, though it should be noted that less older participants were included in the sample. The total daily energy intake of the young adulthood group was higher than the intakes of the middle and older adulthood groups, driven by higher intake of macronutrients, including protein, carbohydrates and total fat. 

#### 3.3.2. Assessment of Reliability 

The highest absolute correlations between the DHQ total scores and ASA-24 data were for cereal (r = 0.395, *p* < 0.001), fruit and vegetables (r = 0.436, *p* < 0.001), and total dietary fiber (r = 0.482, *p* < 0.001). The standardized Cronbach’s alpha for the sample was 0.835. 

Internal consistency assessments revealed correlations between DHQ sub-scores of take-away foods and snacks, fat and omega-3 and intakes of discretionary foods, usual total fat, and omega-3 fatty acids, all b < 0.25. 

### 3.4. Principal Component Analysis Dietary Patterns

PCA identified five dietary patterns (Supplementary Figure S1), explaining 42.12% of the total variance (Table 5). Factor 1 was characterized by high factor loadings for ‘Miscellaneous’, ‘Sugar products and dishes’, and ‘Non-alcoholic beverages’ while having negative loadings for ‘Cereals and cereal products’. Factor 2 was characterized by ‘Special dietary foods’, ‘Alcoholic beverages’, and ‘Fats and oils’. Factor 3 was characterized by high factor loadings for ‘Fruit products and dishes’, ‘Fish and seafood products and dishes’, ‘Dairy and meat substitutes’. Factor 4 was characterized by high factor loadings for ‘Milk products and dishes’, and ‘Meat, poultry and game products and dishes’, while having negative loadings for ‘Soup’. Factor 5 was characterized by high factor loadings for ‘Confectionery and cereal/nut/fruit/seed bars’, ‘Cereals and cereal products’, ‘Vegetable products and dishes’, and ‘Non-alcoholic beverages’.

## 4. Discussion

Our study described the use of an online 24-h recall tool for the evaluation of a diet score, the DHQ, derived from a brief screening tool used with a population of plwMS. Our study demonstrated that elements (sub-scores) of the DHQ are appropriate when a study is focused on intakes of fruit and vegetables due to the specific questions about the quantity of intake. A higher DHQ fruit and vegetable score was found to be an indicator of higher intake of fruit and vegetables as confirmed by the 24-h recall dietary assessment. Our study also revealed variations in food intake for grains, fats and oils, fish and seafood, meats, vegetables and discretionary food items which appeared to have been influenced by ‘MS diets’ that have been promoted within the MS community. 

Our construct validity evaluation of the DHQ showed that the fruit and vegetable sub-scores of the DHQ are appropriate to screen for intakes of fruit and vegetables. Our results were consistent with other studies showing that a brief dietary screener had been applied in approximately 70% of studies to assess fruit and/or vegetables intakes [37]. In our study, the fruit and vegetable DHQ sub-scores reflected the variability of fruit and vegetable intake that were self-reported by the participants when compared with self-reported fruit and vegetable intake data derived from the 24-h recalls. The differences in the vegetable and fruit scores between age groups indicated an ability of the DHQ to detect meaningful differences across these demographics which was not evident by sex. However, the high intake of both vegetables and fruit across our sample, as well as a majority female demographic differs from national intake data for the Australian population. Intake in our sample suggested a majority were meeting the recommended intake of vegetables (5 to 6 serves) and fruits (2 or more) while the Australian Bureau of Statistics reports that only 8.7% of Australians achieve the recommended intake for vegetables and 44.8% for fruit. While the proportion achieving the recommendations shifts to 12.8% and 48.3% for vegetables and fruit, respectively, when considering intakes of females only, this does not account for participants in quartiles 2, 3 and 4 who were all reporting intakes in line with recommendations [38]. The higher reported intake of vegetables and fruit in our sample may be reflective of nutrition information to plwMS, whereby many dietary approaches are heavily focused on intake of these food groups due to their antioxidant properties. Further, the reporting of vegetables and fruit using single item screening tools has been shown to be influenced by reporting bias by sex due to the social desirability of these food groups [39].

Our validity assessment also revealed that although three nutrient sub-scores of the DHQ (total fat, omega-3 and dietary fiber) were assessed, the DHQ scores appear to be less suitable for assessing intake of these nutrients within this population. This is likely due to the wide range of food types that contain the nutrients and the tool using a relatively small number of questions. Further, the food item questions do not include information about quantities of intake making the tool less likely to capture accurate nutrient intake data. Correlations were strongest, though still moderate, for dietary fiber and omega-3 fatty acids. The findings for fiber may in part by explained by the strength of the vegetable and fruit correlations and regularity of intake, while the omega-3 correlations are likely due to the limited food types containing this nutrient. Unsaturated fatty acids, with a focus on long chain fatty acids, are also emphasized in dietary approaches for plwMS. Many dietary approaches recommend a low fat or low saturated fat intake providing insight into food sources of these nutrients. One of the longest running MS studies, the basis to the Swank diet, emphasized the importance of total fat intakes [40] but studies since this time have focused on the fatty acid types, often via supplementation [5]. 

Our internal consistency results demonstrated a weak effect of the sub-scores and our findings also show that the DHQ sub-scores are targeted in assessing the overall diet quality for people with RRMS. While studies have used the total DHQ data as indicative of higher versus lower quality of intake, generally [32], they are unable to differentiate between the complex interrelationships that patterns of eating reflect. Despite this, a recent analysis did find an influence of meat intake as was also found in our analysis [32]. The study also used the HOLISM data providing support for the findings from our smaller sample. 

Dietary intake data used for a diet quality score should be appropriate for its purpose. For this reason, the intake data from the 24-h recall assessment were used in this study to examine the dietary patterns of the sample. Among Australian plwMS surveyed previously, 94.3% reported making changes to their diet and 21.2% followed one or more specific diets [7]. As outlined earlier, common dietary patterns for MS include the Swank diet, Wahl’s diet, the OMS approach, and the Mediterranean diet [8,9]. Thus, the findings of our study likely reflect the differences in the patterns of eating commonly available to plwMS such as the avoidance of dairy, grains and/or meat products [41]. These patterns of eating may also be attributed to a bias of the study participants towards the OMS approach as it was affiliated with the initial recruitment to the HOLISM study. However, the evidence for a particular diet for MS management remains inconclusive. Therefore, a dietary model for plwMS based on scientific evidence is required to develop a suitable dietary quality score in relation to the MS diet-disease relationship.

Although a balanced diet, as advised to the general population, is suggested for plwMS, our data-driven dietary pattern analysis revealed that the greatest variation was in the reported intakes of grains, fats and oils, fish and seafood, meats, and vegetables. Our study’s findings of the variability of the diet align with previous data-driven dietary pattern analyses with the Ausimmune study data which also showed variation in food intakes of meat, fish, dairy products and vegetables [42] in relation to MS risk. These findings were also confirmed using principal factor analysis with the AusLong study of people soon after referral for symptoms suggestive of MS to offer greater insights into the diet-disease relationship for MS [43]. Variations in dietary pattern analyses tend to be influenced by the number of food groups used in the analyses and the statistical methods applied; though the variations were similar when applying the food groups used in the national dietary guidelines. Further, dietary pattern methodology suggests that there is no difference using energy unadjusted and adjusted dietary data to generate dietary patterns [44].

A diet quality score may be needed to differentiate between the varied intakes of plwMS at a food group level. Two diet quality scores that were developed for the general population were also used in a study with a sample of plwMS finding limited consistency between the tools [45]. The relationships examined in relation to MS symptoms of fatigue, anxiety and depression were inconsistent for the tools across the time points. This suggests a need for a tool that is suited to the population that it is being applied, to ensure reliability of findings within and between studies.

The application and interpretation of a diet quality score relies on the underlying content of the dietary data. As some nutrients are stored in the body, recommendations for food and nutrient intakes need to be met over time, rather than on a daily basis. Thus, an approximation of the usual dietary intake offers more information for dietary intake than intake on a given day or over a short period of time. Food frequency questionnaires (FFQs) are informative though time intensive, therefore limit participation, retention, and adherence. Given the accuracy of dietary information depends on the level of detail [46], adequate dietary information (such as food quantities) plays a significant role in the psychometric properties of the DHQ. Therefore, the dietary intake data used for a diet quality score should be appropriate for its purpose. Although dietary intake data derived from a 24-h recall tends to be less prone to bias than FFQs [47], it is unable to derive usual intake data due to the day-to-day intake of using a single recall. However, statistical models, such as MSM used here [31], can be used to calculate usual intake using multiple days of intake. 

In the present study, 31% of the participants completed two or more recalls suggesting that using the online ASA-24 was a feasible tool to generate usual dietary intake data for people with RRMS. Given relying on trained interviewers and multiple administrations is expensive and impractical, collecting detailed dietary intake data in large studies using 24-h dietary recall methods can provide greater depth of data by comparison to a screening tool. With the development of technology, study participants can use the online dietary assessment tool by selecting food items, portion sizes and/or frequencies from a drop-down list or entering the missing items in the database for dietary intake data collection. The analyses of the intakes can be automatically performed and compared with recommendations made in the dietary guidelines. These automated processes can help to reduce the cost and labor-intensive process of dietary intake data collection and coding.

To our knowledge, this is the first study to validate the psychometric properties of a diet score against repeated online 24-h recall dietary assessments in people with RRMS. It is also the first to use a data-driven method to explore the dietary patterns in people with RRMS using detailed self-reported dietary intake data. However, there are several limitations. Firstly, the number of participants was small. Reproducing the analysis in a larger population is warranted to determine the applicability of the findings and allow for more in depth dietary pattern analyses. Data was collected from participants with RRMS, and may not be applicable to other MS phenotypes. Due to the cognitive burden created by MS, two recalls were selected as the minimum to represent usual intakes and participants were asked to include a week and weekend day where possible. Not all participants provided those two recalls, yet no significant differences existed in the data. The DHQ diet quality scores were also developed for the general population not an MS population. A diet quality score for MS may need to differentiate between the varied intakes of plwMS at a food group level beyond those of the DHQ. Although the online 24-h recall performed better than the DHQ, both are self-reported and systematic measurement error may have been introduced. Finally, HOLISM participants may be biased in their dietary pattern as many follow a low saturated fat diet without meat or dairy commonly reported to be based around the OMS approach [48].

## 5. Conclusions

In conclusion, the DHQ appears to be suitable for use in studies focused on screening the intake of fruit and vegetables in people with RRMS. Advances in dietary assessment techniques may overcome the challenges for assessing dietary intake to generate high-quality dietary data in people with RRMS. While it may be assumed that cognitive challenges related to MS limit the ability to accurately recall dietary data, it appears that the multiple pass approach used in the ASA-24 was well suited to this potential challenge. However, an evidence-based dietary model focusing on food is required to monitor the quality of the overall diet and to set priorities in the planning of nutritional interventions for plwMS.

## Figures and Tables

**Table 1:** Sociodemographic characteristics of the sub-sample from the Health Outcomes and Lifestyle In a Sample of people with Multiple sclerosis (HOLISM) cohort, presented for the total sample and by country.

	Australia	New Zealand	United Kingdom/Ireland	United States/Canada	Total
	(n = 41)	(n = 9)	(n = 27)	(n = 19)	(n = 96)
Female sex	34 (82.9%)	7 (77.8%)	19 (70.4%)	19 (100.0%)	79 (82.3%)
Age (yr) *	52.7 ± 10.9 *	55.7± 7.2 *	53.0 ± 7.3 *	48.5 ± 8.3 *	52.2 ± 9.3 *
Weight (kg)	65.0 (57.1, 76.2) ^	64.2 ± 12.7 *	60.0 (56.0, 70.9) ^	73.2 (60.0, 78.2) ^	65.0 (57.5, 73.9) ^
BMI (kg/m^2^)	23.6 (21.6, 26.1) ^	22.8 ± 4.4 *	22.7 (19.8, 21.1) ^	24.7 (21.7, 26.2) ^	23.19 (21.1, 25.7) ^
BMI category					
Normal	28 (68.3%)	7 (77.8%)	19 (70.4%)	12 (63.2%)	66 (68.8%)
Overweight	9 (22.0%)	1 (11.1%)	5 (18.5%)	5 (26.3%)	20 (20.8%)
Obese	4 (9.8%)	1 (11.1%)	3 (11.1%)	2 (10.5%)	10 (10.4%)
MS phenotype at baseline					
Benign ^a^	4 (9.8%)	1 (11.1%)	1 (3.7%)	0 (0.0%)	6 (6.3%)
Relapsing-remitting	31 (75.6%)	7 (77.8%)	23 (85.2%)	16 (84.2%)	77 (80.2%)
Secondary-progressive	1 (2.4%)	0 (0.0%)	0 (0.0%)	0 (0.0%)	1 (1.0%)
Unsure/other	5 (12.2%)	1 (11.1%)	3 (11.1%)	3 (15.8%)	12 (12.5%)
Duration since diagnosis (years)	4.4 (2.3, 9.0) ^	4.4 ± 2.5 *	2.4 (2.4, 11.0) ^	2.4 (1.4, 11.4) ^	3.9 (1.8, 8.6) ^
Duration since onset (years)	8.6 (4.2, 17.4) ^	8.5 ± 6.0 *	7.4 (4.4, 20.5) ^	10.3 (6.5, 14.4) ^	8.5 (4.4, 17.1) ^

* Mean ± SD. BMI—Body mass index, ^ Median (interquartile range). ^a^ Referring to stable phase of relapsing remitting multiple sclerosis. MS: Multiple sclerosis.

**Table 2:** Intakes of nutrients and food groups of the sub-sample from the Health Outcomes and Lifestyle In a Sample of people with Multiple sclerosis (HOLISM) cohort, presented for the total sample and by country.

	Australia	New Zealand	United Kingdom/Ireland	United States/Canada	Total
	(n = 41)	(n = 9)	(n = 27)	(n = 19)	(n = 96)
Diet Habits Questionnaire total score	84.5 (75.8, 88.1) ^	88.5 ± 9.3 *	87.5 ± 8.1 *	79.2 ± 11.4 *	85.5 (77.04, 91.83) ^
ASA-24 Food groups					
Cereals (serves)	4.7 ± 1.9 *	5.0 ± 1.7 *	6.4 ± 2.4 *	5.4 ± 2.0 *	5.4 ± 2.1 *
Vegetables (serves)	6.5 (4.3, 10) ^	8.4 (5.0, 10.2) ^	6.6 (4.7, 9.5) ^	6.1 (4.2, 8.1) ^	6.5 (4.5, 9.6) ^
Fruits (serves)	1.5 (0.8, 2.7) ^	2.1 (1.0, 3.3) ^	2.5 (1.5, 3.4) ^	1.5 (0.8, 2.7) ^	1.8 (1.0, 2.8) ^
Milk and alternatives (serves)	0.9 (0.4, 1.7) ^	0.5 (0.3, 0.9) ^	0.5 (0.4, 1.5) ^	0.9 (0.5, 1.6) ^	0.8 (0.4, 1.6) ^
Meat and alternatives (serves)	2.5 (1.8, 3.3) ^	2.6 (1.6, 3.2) ^	1.9 (1.5, 2.7) ^	2.3 (1.5, 2.6) ^	2.2 (1.6, 3.0) ^
Discretionary foods (serves)	3.0 (1.7, 4.2) ^	2.8 (1.7, 4.4) ^	2.4 (1.4, 3.4) ^	1.6 (1.3, 1.9) ^	2.2 (1.4, 3.8) ^
ASA-24 Total daily energy intake (MJ)	8.0 ± 2.1 *	7.7 ± 1.8 *	8.5 ± 2.1 *	7.9 ± 1.8 *	8.1 ± 2.0 *
ASA-24 Nutrients					
Protein (g)	73.5 (54.1, 91.9) ^	73.0 ± 15.6 *	80.0 ± 23.0 *	72.6 ± 21.5 *	75.2 (57.9, 93.3) ^
Carbohydrate (g)	199.4 ± 74.5 *	201.9 ± 47.5 *	243.4 ± 74.3 *	234.9 ± 74.5 *	219.1 ± 74.2 *
Total Fat (g)	72.0 (61.0, 89.2) ^	62.8 (53.0, 71.3) ^	69.9 ± 20.7 *	68.2 ± 17.4 *	67.5 (58.3, 82.7) ^
Fatty acids, total saturated (g)	18.0 (13.4, 25.0) ^	14.1 ± 3.0 *	16.0 ± 7.1 *	16.0 (10.7, 20.8) ^	15.8 (12.1, 21.6) ^
Fatty acids, total monounsaturated (g)	28.9 (24.4, 38.9) ^	24.8 (20.0, 28.8) ^	27.3 (23.3, 32.3) ^	26.0 ± 6.8 *	27.3 (23.3, 32.7) ^
Fatty acids, total polyunsaturated (g)	17.4 (14.9, 22.5) ^	17.4 ± 7.3 *	17.9 ± 6.6 *	18.4 ± 6.1 *	17.0 (14.5, 22.3) ^
Omega fatty acids (EPA+DPA+DHA) (mg)	133.1 (63.8, 897.3) ^	131.9 (64.7, 710.8) ^	108.0 (32.0, 471.1) ^	61.6 (31.8, 484.1) ^	113.0 (34.8, 480.8) ^
Sugars, total (g)	79.9 (50.3, 110.1) ^	79.2 (62.8, 102.5) ^	97.9 ± 39.6 *	84.8 ± 30.9 *	87.6 (59.7, 110.6) ^
Fiber, total dietary (g)	33.0 ± 13.7 *	37.9 ± 16.9 *	44.5 ± 18.9 *	29.6 ± 10.4 *	34.1 (24.7, 46.6) ^
Alcohol (g)	2.2 (0.7, 10.4) ^	10.8 (0.9, 23.5) ^	2.1 (0.8, 12.9) ^	1.4 (0.8, 15.6) ^	2.2 (0.8, 14.3) ^
Calcium (mg)	775.0 ± 318.1 *	652.1 ± 165.0 *	849.9 ± 451.6 *	824.3 (531.4, 1159.5) ^	782.4 (526.0, 996.0) ^
Iron (mg)	14.0 ± 4.3 *	13.7 ± 4.7 *	16.3 ± 5.3 *	17.1 ± 6.2 *	15.2 (11.1, 18.5) ^
Magnesium (mg)	428.1 ± 139.3 *	504.7 ± 167.9 *	506.6 ± 174.2 *	390.0 ± 117.7 *	449.8 ± 153.6 *
Potassium (mg)	3504.9 ± 1271.2 *	3817.8 ± 1175.2 *	3975.5 ± 1434.2 *	3056.4 ± 796.8 *	3577.8 ± 1259.8 *
Sodium (mg)	2409.6 ± 870.2 *	2099.8 ± 434.1 *	2335.4 ± 745.0 *	3055.9 ± 886.2 *	2359.8 (1903.5, 3060.3) ^
Zinc (mg)	8.9 (7.1, 11.5) ^	9.7 ± 2.2 *	10.3 ± 3.1 *	9.6 (7.2, 10.9) ^	9.6 (7.4, 11.5) ^
Vitamin C (mg)	122.4 (56.1, 163.9) ^	226.2 ± 113.9 *	137.6 (91.0, 208.0) ^	88.6 (52.4, 205.3) ^	132.2 (64.4, 191.4) ^
Thiamin (mg)	1.4 ± 0.5 *	1.3 ± 0.4 *	1.6 ± 0.5 *	1.7 (1.2, 2.2) ^	1.5 (1.1, 1.8) ^
Riboflavin (mg)	1.6 ± 0.7 *	1.2 (1.1, 1.7)	1.6 ± 0.8 *	1.8 (1.5, 2.7) ^	1.7 (1.0, 2.2) ^
Folate, total (µg)	503.5 ± 146.6 *	565.7 ± 201.9 *	561.4 ± 171.9 *	535.0 (411.9, 646.5) ^	494.1 (420.5, 656.0) ^
Vitamin B-12 (µg)	3.4 (1.5, 4.6) ^	2.3 ± 0.9 *	2.5 (1.4, 3.2) ^	3.0 (1.6, 5.6) ^	2.7 (1.6, 4.0) ^
Retinol equiv (µg)	1032.2 (908.5, 1742.7) ^	1071.7 (751.1, 1646.7) ^	1317.1 ± 653.2 *	882.1 (664.6, 1175.9) ^	1048.8 (803.4, 1588.2) ^
Beta-carotene (µg)	4902.9 (3538.9, 7752.1) ^	4767.7 (3211.5, 8008.1) ^	5946.4 ± 3056.0 *	5626.3 (3190.2, 7442.4) ^	4934.3 (3438.5, 7456.6) ^

* Mean ± SD, ^ Median (interquartile range).

**Table 3:** Usual daily dietary intake by quartiles of Diet Habit Questionnaire.

	Q1 (n = 24)	Q2 (n = 24)	Q3 (n = 24)	Q4 (n = 24)	*p*-Value for Trend ^a^
**Diet Habits Questionnaire score**					
Total	68.4 ± 5.7 *	81.4 ± 2.8 *	88.6 ± 2.0 *	94.7 ± 2.3 *	**<0.01**
Cereal	3.0 (3.0, 3.0) ^	3.5 (3.0, 4.0) ^	3.75 (3.0, 4.0) ^	4.5 (4.0, 5.0) ^	**<0.01**
Fruit and Vegetables	3.0 (2.5, 3.0) ^	3.5 (3.0, 4.0) ^	4.00 (3.6, 4.4) ^	4.50 (4.0, 4.5) ^	**<0.01**
Limit take-away foods	2.5 (2.0, 3.0) ^	3.0 (2.5, 3.5) ^	3.00 (3.0, 3.5) ^	3.50 (3.5, 3.5) ^	**<0.01**
Fiber	3.0 (2.5, 3.4) ^	2.5 (3.1, 4.0) ^	4.00 (3.5, 4.0) ^	4.50 (4.1, 4.5) ^	**<0.01**
Fat	3.0 (3.0, 3.0) ^	3.5 (3.5, 4.0) ^	4.00 (4.0, 4.0) ^	4.00 (4.0, 4.5) ^	**<0.01**
Omega-3	3.0 (2.0, 4.0) ^	4.0 (2.0, 4.0) ^	5.00 (4.0, 5.0) ^	4.50 (4.0, 5.0) ^	**<0.01**
Food choices	3.5 (2.6, 3.5) ^	4.3 (3.6, 4.5) ^	4.50 (4.5, 5.0) ^	4.50 (4.5, 5.0) ^	**<0.01**
Food preparation	3.5 (2.6, 3.5) ^	4.3 (3.6, 4.5) ^	4.50 (4.5, 5.0) ^	4.50 (4.5, 5.0) ^	**<0.01**
**ASA-24 Food groups**					
Cereals (serves)	4.5 ± 2.2 *	5.9 ± 2.6 *	5.1 ± 1.4 *	6.00 ± 2.1 *	0.055
Vegetables (serves)	3.9 (2.5, 6.9) ^	7.1 (4.3, 9.0) ^	6.5 (5.1, 9.6) ^	9.0 (5.6, 10.8) ^	**<0.01**
Fruits (serves)	1.3 (0.5, 2.) ^	1.5 (0.9, 3.0) ^	2.1 (1.0, 2.8) ^	2.5 ± 1.1 *	0.141
Milk and alternatives (serves)	1.2 (0.4, 1.8) ^	1.0 ± 0.7 *	0.58 (0.3, 1.4) ^	0.7 (0.5, 1.5) ^	0.210
Meat and alternatives (serves)	1.9 (1.3, 2.5) ^	2.3 ± 1.3 *	2.6 (1.7, 3.1) ^	2.6 ± 1.1 *	0.274
Discretionary foods (serves)	2.0. (1.1, 3.8) ^	2.5 ± 1.9 *	2.4 (1.9, 4.3) ^	2.5 ± 1.3 *	0.647
**ASA-24 Energy (MJ)**	7.5 ± 2.5 *	8.3 ± 2.0 *	8.1 ± 1.8 *	8.6 ± 1.6 *	0.331
**ASA-24 Nutrients**					
Protein (g)	60.40 (44.4, 84.1) ^	77.0 ± 27.0 *	78.3 ± 25.0 *	81.9 ± 18.8 *	0.397
Carbohydrate (g)	199.1 ± 87.7 *	225.4 ± 81.9 *	219.0 ± 62.5 *	232.8 ± 61.5 *	0.440
Total Fat (g)	70.5 ± 21.2 *	76.5 ± 19.4 *	68.3 (58.2, 75.4) ^	67.4 (61.6, 71.7) ^	0.592
Fatty acids, total saturated (g)	16.9 (14.4, 32.5) ^	18.1 ± 7.5 *	15.5 ± 5.1 *	15.3 ± 4.9 *	**<0.01**
Fatty acids, total monounsaturated (g)	28.1 ± 8.8 *	30.1 (23.9, 41.3) ^	26.8 (21.9, 30.4) ^	26.2 (23.4, 29.2) ^	0.547
Fatty acids, total polyunsaturated (g)	14.6 ± 4.8 *	18.3 (15.0, 23.6) ^	18.9 ± 7.0 *	20.0 ± 5.7 *	**<0.01**
Omega fatty acids (EPA+DPA+DHA) (mg)	110.6 (32.6, 408.3) ^	62.7 (34.4, 278.6) ^	194.6 (56.9, 831.1) ^	190.0 (37.4, 1360.3) ^	0.124
Sugars, total (g)	71.5 (45.1, 112.9) ^	78.6 (59.7, 106.1) ^	93.8 ± 39.4 *	93.0 ± 29.8 *	0.711
Fiber, total dietary (g)	23.5 ± 10.7 *	36.8 ± 14.8 *	35.5 ± 11.9 *	48.2 ± 16.2 *	**<0.01**
Alcohol (g)	1.5 (0.4, 11.7) ^	1.49 (0.9, 2.8) ^	2.5 (1.0, 14.2) ^	2.3 (0.8, 26.4) ^	0.066
Calcium (mg)	804.0 ± 459.8 *	750.7 ± 301.8 *	777.1 (509.0, 922.2) ^	927.3 ± 365.3 *	0.403
Iron (mg)	12.0 (8.3, 14.6) ^	16.1 ± 5.5 *	15.1 ± 4.8 *	17.5 ± 4.2 *	**<0.01**
Magnesium (mg)	333.6 ± 125.9 *	447.8 ± 134.7 *	458.2 ± 129.4 *	559.6 ± 141.4 *	**<0.01**
Potassium (mg)	2751.7 ± 1078.2 *	3356.1 (2498.3, 4218.8) ^	3395.9 (3062.3, 4094.7) ^	4287.1 ± 978.6 *	**<0.01**
Sodium (mg)	2517.2 ± 1030.6 *	2536.0 ± 870.8 *	2446.6 ± 764.1 *	2450.7 ± 760.0 *	0.977
Zinc (mg)	7.8 (6.6, 11.0) ^	9.9 ± 3.4 *	9.2 (7.9, 11.5) ^	10.7 ± 2.4 *	0.696
Vitamin C (mg)	67.8 (46.1, 150.1) ^	90.4 (55.1, 201.2) ^	139.3 (106.8, 180.6) ^	190.3 (133.6, 278.5) ^	**<0.01**
Thiamine (mg)	1.2 ± 0.5 *	1.5 ± 0.6 *	1.3 (1.1, 1.8) ^	1.8 ± 0.6 *	**0.021**
Riboflavin (mg)	1.5 (0.9, 1.8) ^	1.6 ± 0.7 *	1.6 (1.0, 2.3) ^	1.8 ± 0.7 *	0.772
Folate, total (mcg)	426.6 ± 127.9 *	542.8 ± 163.4 *	487.1 (413.1, 663.9) ^	613.2 ± 132.3 *	**<0.01**
Vitamin B-12 (mcg)	2.6 (1.7, 3.9) ^	2.9 (1.2, 3.9) ^	3.3 (1.2, 4.8) ^	2.5 (1.60, 4.1) ^	0.771
Vitamin A (mcg)	1033.9 ± 569.6 *	1147.1 (825.5, 1684.7) ^	1041.0 (865.8, 1564.4) ^	1338.0 ± 632.8 *	0.370
Beta-carotene (mcg)	3479.4 (2598.4, 7023.1) ^	5597.2 (3615.8, 7905.3) ^	4888.2 (4235.2, 7786.7) ^	5712.36 (3754.5, 8701.5) ^	0.420

^a^ Linear regression, * Mean ± SD, ^ Median (interquartile range). Statistically signficant findings highlighted in bold.

**Table 4:** Diet Habit Questionnaire scores and ASA-24 intake of food groups and nutrients, by sex and age.

	Sex			Age Group			
	Female (n = 79)	Male (n = 17)	*p*-Value	33–44 y (n = 20)	45–64 y (n = 67)	65–86 y (n = 9)	*p*-Value ^#^
**Diet Habits Questionnaire score**							
Total	85.5 (76.0, 91.8)	85.0 (79.6, 90.5)	0.943 ^	79.6 ± 9.3	83.9 ± 10.7	86.6 ± 10.0	0.163
Cereal	3.5 (3.0, 4.5)	4.0 (3.0, 4.3)	0.370 ^	3.6 ± 0.7	3.8 ± 0.8	3.7 ± 0.8	0.689
Fruit and Vegetables	4.0 (3.0, 4.0)	3.5 (3.0,4.0)	0.296 ^	3.4 ± 0.8	3.7 ± 0.7	4.1 ± 0.3	**<0.05**
Limit take-away foods	3.0 (2.5, 3.5)	3.0 (2.5, 3.5)	0.627 ^	2.9 ± 0.7	2.9 ± 0.6	3.3 ± 0.4	0.231
Fiber	4.0 (3.0, 4.5)	3.5 (3.0, 4.0)	0.449 ^	3.4 ± 0.7	3.7 ± 0.7	4.1 ± 0.3	**<0.05**
Fat	4.0 (3.0, 4.0)	4.0 (3.5, 4.0)	0.829 ^	3.5 ± 0.5	3.7 ± 0.5	3.8 ± 0.6	0.251
Omega-3	4.0 (2.0, 5.0)	5.0 (4.0, 5.0)	<0.05 ^	3.4 ± 1.2	3.6 ± 1.5	4.4 ± 0.5	0.132
Food choices	4.0 (3.5, 4.5)	4.5 (4.25, 5.0)	<0.05 ^	3.8 ± 1.0	4.2 ± 0.8	4.3 ± 0.8	0.156
Food preparation	4.0 (3.5, 4.5)	4.5 (4.25, 5.0)	<0.05 ^	3.8 ± 1.0	4.2 ± 0.8	4.3 ± 0.8	0.156
**ASA-24 Food groups**							
Cereals (serves)	5.0 ± 1.9	7.1 ± 2.3	<0.01 *	5.9 ± 2.0	5.4 ± 2.2	4.2 ± 1.7	0.118
Vegetables (serves)	6.5 (4.2, 9.5)	8.1 (5.2, 10.5)	0.217 ^	7.2 ± 3.0	7.5 ± 4.6	8.4 ± 3.7	0.778
Fruits (serves)	1.7 (1.0, 2.7)	2.8 (1.1, 3.6)	0.100 ^	1.5 ± 1.2	2.3 ± 1.7	2.0 ± 0.8	0.154
Milk and alternatives (serves)	1.0 (0.5, 1.7)	0.5 (0.2, 0.8)	0.077 ^	1.2 ± 1.1	1.1 ± 0.9	0.9 ± 0.6	0.534
Meat and alternatives (serves)	2.1 (1.6, 2.8)	3.0 (1.9, 4.3)	<0.01 ^	2.5 ± 1.7	2.4 ± 1.1	2.4 ± 1.4	0.901
Discretionary foods (serves)	2.2 (1.4, 3.4)	3.0. (1.8, 5.5)	0.070 ^	2.1 ± 1.6	2.8 ± 2.0	2.6 ± 1.2	0.322
**ASA-24 Energy (MJ)**	7.8 ± 1.7	10.1 ± 2.0	<0.01 *	8.7 ± 1.6	8.1 ± 2.1	7.4 ± 1.8	0.270
**ASA-24 Nutrients**							
Protein (g)	71.9 ± 22.1	98.1 ± 35.3	<0.01 *	87.9 ± 33.5	73.7 ± 22.4	72.1 ± 35.0	0.099
Carbohydrate (g)	210.8 ± 73.0	257.4 ± 69.4	<0.05 *	221.2 ± 63.9	224.4 ± 78.1	174.5 ± 53.5	0.165
Total Fat (g)	67.5 ± 15.9	91.4 ± 24.0	<0.01 *	81.4 ± 15.9	69.0 ± 20.2	70.9 ± 18.7	**0.045**
Fatty acids, total saturated (g)	15.3 (11.9, 21.5)	19.7 (14.2, 24.1)	0.141 ^	23.0 ± 9.5	16.5 ± 7.4	16.3 ± 4.4	**<0.01**
Fatty acids, total monounsaturated (g)	26.2 (22.5, 30.2)	40.9 (29.6, 51.9)	<0.01 ^	32.7 ± 8.1	28.2 ± 10.1	31.9 ± 10.1	0.137
Fatty acids, total polyunsaturated (g)	16.7 (14.5, 21.6)	21.3 (15.4, 27.7)	0.073 ^	19.0 ± 4.7	18.2 ± 7.0	17.7 ± 3.8	0.854
Omega fatty acids (EPA+DPA+DHA) (mg)	108.0 (34.1, 484.1)	308.9 (46.1, 699.4)	0.406 ^	663.4 ± 1168.8	437.3 ± 753.5	508.1 ± 557.0	0.575
Sugars, total (g)	89.9 (54.8, 113.7)	83.1 (72.7, 102.9)	0.608 ^	78.8 ± 28.8	93.2 ± 42.5	78.3 ± 21.4	0.245
Fiber, total dietary (g)	32.2 (24.2, 44.3)	46.59 (29.9, 53.1)	<0.05 ^	34.2 ± 11.4	36.7 ± 17.3	35.0 ± 15.4	0.826
Alcohol (g)	1.6 (0.7, 10.8)	2.3 (1.0, 22.7)	0.151 ^	8.2 ± 15.5	9.6 ± 14.2	9.2 ± 17.6	0.930
Calcium (mg)	802.5 (581.3, 1024.2)	603.1 (452.6, 859.6)	0.174 ^	875.6 ± 400.8	816.3 ± 400.3	668.5 ± 156.7	0.411
Iron (mg)	14.5 (10.9, 16.9)	18.0 (13.7, 20.0)	0.061 ^	17.3 ± 5.5	14.8 ± 5.0	14.0 ± 4.5	0.113
Magnesium (mg)	431.7 ± 148.3	533.7 ± 154.4	<0.05 *	407.0 ± 95.0	460.8 ± 167.5	463.1 ± 147.0	0.379
Potassium (mg)	3399.1 ± 1170.5	4408.4 ± 1361.3	<0.01 *	3313.6 ± 942.8	3590.5 ± 1330.2	4071.0 ± 1297.1	0.325
Sodium (mg)	2293.9 (1846.4, 2975.6)	2681.8 (2233.0, 3165.6)	0.069 ^	2799.7 ± 851.6	2406.9 ± 845.3	2394.7 ± 816.8	0.183
Zinc (mg)	9.2 (7.3, 11.3)	11.4 (8.4, 14.0)	0.029 ^	11.6 ± 5.6	9.6 ± 3.1	10.3 ± 5.8	0.149
Vitamin C (mg)	132.7 (60.1, 191.7)	114.6 (76.6, 197.1)	0.905 ^	115.8 ± 66.2	154.4 ± 107.5	181.8 ± 109.9	0.195
Thiamin (mg)	1.4 (1.1, 1.7)	1.9 (1.1, 2.3)	0.300 ^	1.6 ± 0.5	1.5 ± 0.78	1.4 ± 0.6	0.848
Riboflavin (mg)	1.7 (1.0, 2.2)	1.4 (1.0, 2.1)	0.561 ^	1.9 ± 0.9	1.6 ± 0.8	1.9 ± 0.6	0.351
Folate, total (µg)	481.0 (411.0, 634.0)	683.5 (527.3, 753.1)	<0.01 ^	526.2 ± 118.0	534.8 ± 178.3	574.6 ± 200.2	0.767
Vitamin B-12 (µg)	2.6 (1.5, 4.0)	2.9 (1.8, 4.6)	0.542 ^	4.7 ± 3.2	2.8 ± 2.0	3.9 ± 4.1	**<0.01**
Retinol (µg)	1032.2 (801.1, 1578.3)	1288.1 (799.7, 1756.8)	0.385 ^	1245.5 ± 573.4	1140.9 ± 541.3	1951.3 ± 1148.0	**<0.01**
Beta-carotene (µg)	4894.6 (3505.2, 7461.3)	5838.5 (3404.9, 7472.8)	0.712 ^	5762.5 ± 2689.0	5353.1 ± 2612.1	9271.6 ± 6359.7	**<0.01**

* Independent-samples T test, ^ Wilcoxon-Mann–Whitney test, # One-Way ANOVA with Bonferroni. Statistically signficant findings highlighted in bold.

**Table 5:** Factor loadings for food groups that loaded highly (|>0.2|) in varimax rotated principal components.

Factor	Food Group by Weight in Grams (Factor Loading)	Eigenvalue	% of Variance (Total: 42.12%)
1	Miscellaneous ^a^ (0.82) Sugar products and dishes (0.81) Non-alcoholic beverages (0.20) Cereals and cereal products (−0.20)	2.19	10.42
2	Special dietary foods ^b^ (0.81) Alcoholic beverages (0.73) Fats and oils (0.62)	1.80	8.57
3	Fruit products and dishes (0.63) Fish and seafood products and dishes (0.59) Dairy and meat substitutes (0.52)	1.72	8.19
4	Milk products and dishes (0.79) Meat, poultry and game products and dishes (0.46) Soup (−0.28)	1.69	8.05
5	Confectionery and cereal/nut/fruit/seed bars (0.84) Cereals and cereal products (0.63) Vegetable products and dishes (0.44) Non-alcoholic beverages (0.41)	1.45	6.89

Factor loadings ≥2 were selected, each food group has high or low or meaningful attribution to one factor only, ^a^ Includes yeast and yeast and vegetable or meat extracts; Intense sweeteners; Herbs, spices, seasonings and stock cubes; Essences; and Chemical raising agents and cooking ingredient food groups. ^b^ Includes vitamin and mineral supplements; oil supplements; herbal and homeopathic supplements; other non-nutritive supplements; and other medicines.

## Data Availability

The data presented in this study are available on request from the corresponding author. The data are not publicly available due to clinical sensitivity of the information.

## Supplementary Materials

The following supporting information can be downloaded at: https://www.mdpi.com/article/10.3390/nu14214568/s1, Table S1: Australian Food, Supplement, and Nutrient Database for Estimation of Population Nutrient Intakes 2011–2013 major food groups and examples; Figure S1: Scree plot from principal components analysis of major food groups; Figure S2: Participants flow diagram.
