# Supplementary material for: Using Online 24-h Dietary Methodology to Validate the Psychometric Properties of a Dietary Scoring Tool with an International Sample of Adults Living with Multiple Sclerosis
Source: Nutrients. 2022 Oct 31;14(21):4568. doi: 10.3390/nu14214568 (PMC9654468; doi:10.3390/nu14214568)
Supplement: Supplementary file 1 [file nutrients-14-04568-s001.zip › nutrients-1797151-supplementary.pdf]

Supplementary Table S1: Australian Food, Supplement, and Nutrient Database for Estimation of Population Nutrient Intakes 2011-2013 major food groups and examples

Major food groups	Examples
Alcoholic beverages	Beers, wines, spirits, cocktails, liqueurs
Cereal based products and dishes	Biscuits, cakes, muffins, pastries, pizza, burger, pancakes
Cereals and cereal products	Bread, breakfast cereals, noodles, pasta, rice
Confectionery and cereal/nut/fruit/seed bars	Chocolate, muesli bar, fruit bar, lollies, chewing gum
Dairy & meat substitutes	Soy beverages, tofu, tofu stir fry
Egg products and dishes	Eggs, egg dishes such as scrambled eggs, omelet, mousse
Fats and oils	Butter, margarine, oils
Fish and seafood products and dishes	Fresh fin fish, frozen fin fish, smoked fish, canned fish, prawn, squid, fish cake, tuna mornay with cheese, garlic prawn
Fruit products and dishes	Apple, pear, berries, oranges, peaches, banana, melon, dried fruit, apple crumble
Legume and pulse products and dishes	Soy beans, chickpeas, kidney beans, lentils, falafel, dhal
Meat, poultry and game products and dishes	Beef, veal, lamb, pork, chicken, sausage, bacon, ham, dried meats, crumbled meats, meat bolognaise pasta sauce, casserole, curries
Milk products and dishes	Milk, yoghurt, cream, cheese, ice cream, rice pudding, cheese cake
Miscellaneous	Yeast, salt, intense sweeteners, herbs, stock, essences, gelatin, spreadable yeast extract
Non-alcoholic beverages	Coffee, tea, fruit juice, cordial, soft drink, water, electrolyte drinks
Savoury sauces and condiments	Gravies, savoury sauces, pickles, salad dressing
Seed and nut products and dishes	Pumpkin seeds, linseed, sesame seed, peanuts, peanut butter, almond, coconut milk
Snack foods	Potato crisps, popcorn, corn chips, pretzels
Soup	Canned and homemade soup, dried soup mix
Special dietary foods	Liquid and powdered meal replacements, protein drinks and powders, oral supplement powder and beverages
Sugar products and dishes	Sugar, honey, jam, icing sugar, apple sauce, meringue
Vegetable products and dishes	Potatoes, cabbage, carrots, lettuce, beans, fresh herbs, tomato, pumpkin, sweetcorn, onion, salad

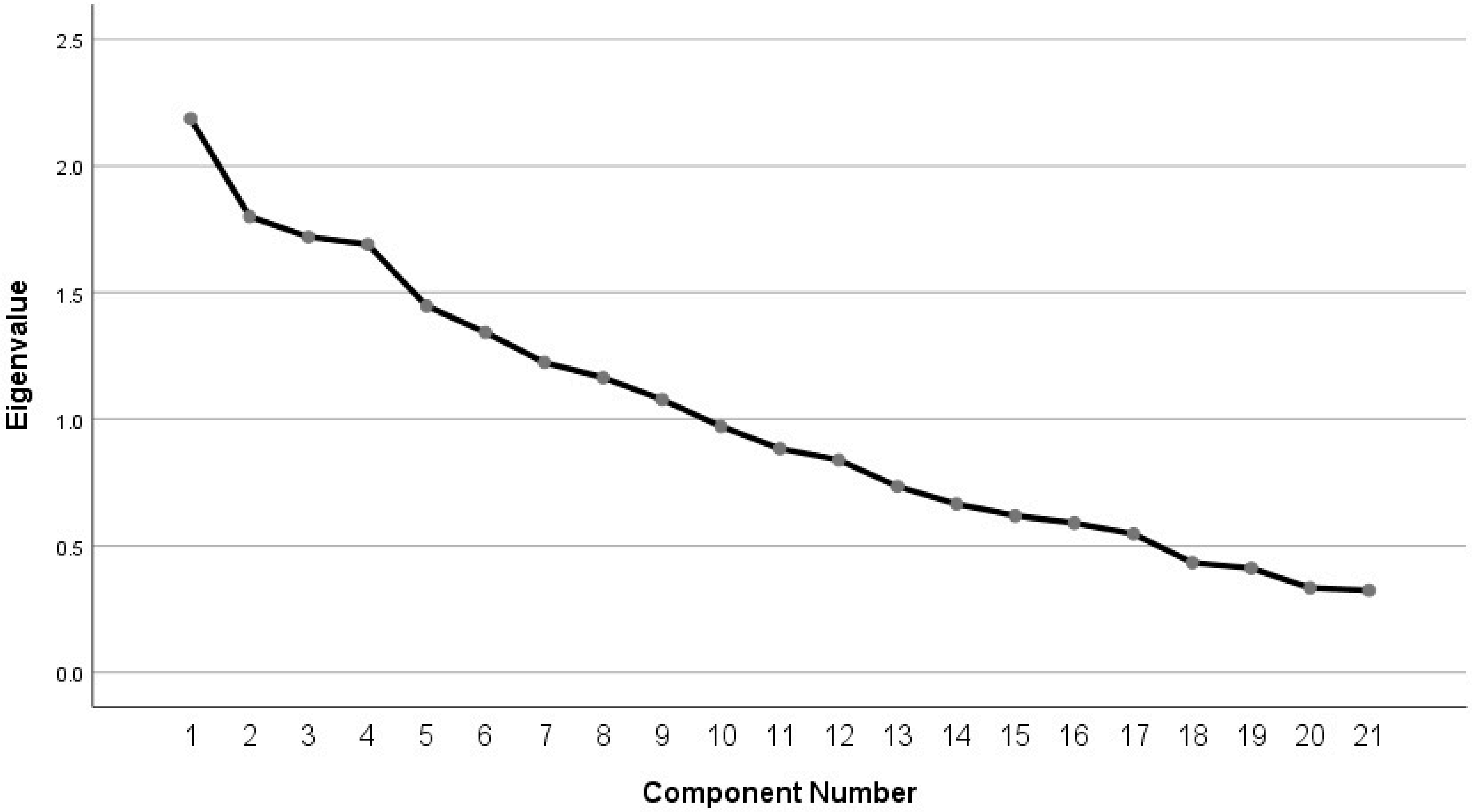

Supplementary Figure S1: Scree plot from principal components analysis of major food groups

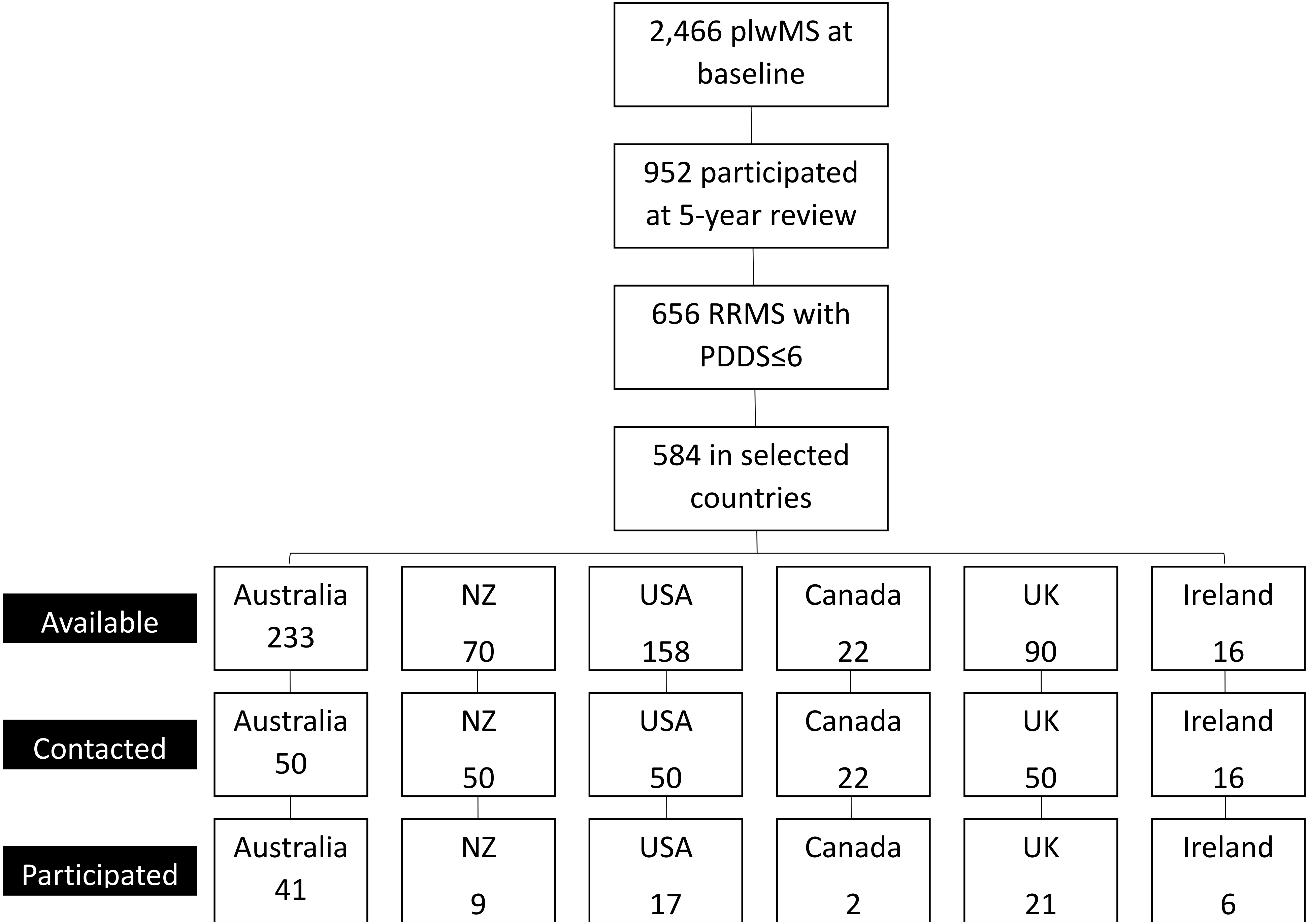

Supplementary Figure S2: Participants flow diagram
Abbreviations: MS: multiple sclerosis, pwlMS Person living with MS, RRMS: relapsing remitting MS, PDDS: patient determined disease steps, NZ: New Zealand, USA: United States of America, UK: United Kingdom
